# Ultrasound mapping of pelvic endometriosis: does the location and number of lesions affect the diagnostic accuracy? a multicentre diagnostic accuracy study

**DOI:** 10.1186/1472-6874-13-43

**Published:** 2013-10-29

**Authors:** Tom K Holland, Alfred Cutner, Ertan Saridogan, Dimitrios Mavrelos, Kate Pateman, Davor Jurkovic

**Affiliations:** 1 Early Pregnancy and Gynaecology Assessment Unit, Department of Obstetrics and Gynaecology, Suite 8, Golden Jubilee Wing, King’s College Hospital, London SE5 8RX, UK; 2 Department of Obstetrics and Gynaecology, University College Hospital, 235 Euston Road, London NW1 2BU, UK

## Abstract

**Background:**

Endometriosis is a common condition which causes pain and reduced fertility. Treatment can be difficult, especially for severe disease, and an accurate preoperative assessment would greatly help in the managment of these patients. The objective of this study is to assess the accuracy of pre-operative transvaginal ultrasound scanning (TVS) in identifying the specific features of pelvic endometriosis and pelvic adhesions in comparison with laparoscopy.

**Methods:**

Consecutive women with clinically suspected or proven pelvic endometriosis, who were booked for laparoscopy, were invited to join the study. They all underwent a systematic transvaginal ultrasound examination in order to identify discrete endometriotic lesions and pelvic adhesions. The accuracy of ultrasound diagnosis was determined by comparing pre-operative ultrasound to laparoscopy findings.

**Results:**

198 women who underwent preoperative TVS and laparoscopy were included in the final analysis. At laparoscopy 126/198 (63.6%) women had evidence of pelvic endometriosis. 28/126 (22.8%) of them had endometriosis in a single location whilst the remaining 98/126 (77.2%) had endometriosis in two or more locations. Positive likelihood ratios (LR+) for the ultrasound diagnosis of ovarian endometriomas, moderate or severe ovarian adhesions, pouch of Douglas adhesions, and bladder deeply infiltrating endometriosis (DIE), recto-sigmoid colon DIE, rectovaginal DIE, uterovesical fold DIE and uterosacral ligament DIE were >10, whilst for pelvic side wall DIE and any ovarian adhesions the + LH was 8.421 and 9.81 respectively.

The negative likelihood ratio (LR-) was: <0.1 for bladder DIE; 0.1-0.2 for ovarian endometriomas, moderate or severe ovarian adhesions, and pouch of Douglas adhesions; 0.5-1 for rectovaginal, uterovesical fold, pelvic side wall and uterosacral ligament DIE. The accuracy of TVS for the diagnosis of both total number of endometriotic lesions and DIE lesions significantly improved with increasing total number of lesions.

**Conclusions:**

Our study has shown that the TVS diagnosis of endometriotic lesion is very specific and false positive results are rare. Negative findings are less reliable and women with significant symptoms may still benefit from further investigation even if TVS findings are normal. The accuracy of ultrasound diagnosis is significantly affected by the location and number of endometriotic lesions.

## Background

Endometriosis is a common gynaecological condition, defined as the presence of endometrial-like tissue outside the uterus, which impairs quality of life. In more severe cases it forms cysts in the ovaries and deeply infiltrates pelvic organs.

In women with pelvic endometriosis ultrasound examination has been shown to be able to diagnose ovarian endometriomas with a high degree of accuracy, but other endometriotic lesions were considered to be undetectable [1]. Several recent studies have shown that a targeted ultrasound examination using high resolution equipment can also detect deep infiltrating endometriotic (DIE) lesions, which are affecting other organs within the lesser pelvis. The reported accuracy of the ultrasound diagnosis of DIE varies between different studies, which may reflect the variations in the examination technique, quality of ultrasound equipment and experience of the operators. The prevalence of disease is also variable in different studies, which may bias the findings. Only a few studies have attempted to assess the ability of ultrasound examination to detect presence of pelvic adhesions in women with pelvic endometriosis and to assess their severity [2,3].

The detection of all endometriotic lesions within the pelvis and assessment of the severity of adhesions are required in order to assess the severity of endometriosis using the standard revised ASRM classification and triage women for surgical treatment. Women with severe disease and extensive adhesions could be thus referred to centres of excellence to ensure complete surgical excision [4].

The objective of this study is to assess the accuracy of pre-operative transvaginal ultrasound scanning (TVS) in identifying the specific features of pelvic endometriosis and pelvic adhesions in comparison to laparoscopy.

## Methods

This was a prospective, observational, multicentre study, which was conducted at King’s College Hospital and University College Hospital in London. These are both major teaching hospitals and the latter has a specialist tertiary referral endometriosis centre. Consecutive women with clinically suspected or proven pelvic endometriosis were invited to join the study. The inclusion criteria were: pre-menopausal women with a clinical suspicion of endometriosis awaiting diagnostic laparoscopy or women diagnosed with pelvic endometriosis at diagnostic laparoscopy awaiting operative treatment. Other criteria included age 16 or over and the ability to provide informed consent. Women who could not undergo a transvaginal ultrasound scan and those who became pregnant whilst awaiting surgery were excluded from the study.

The study was ethically approved and an information leaflet was given to all eligible women before assessment. Informed consent was obtained from all women who agreed to take part in the study.

### Procedures

All women were assessed by the attending clinicians who obtained a detailed history, which was recorded on a dedicated clinical database (ViewPoint, GE Healthcare, Fairfield, Connecticut, USA). Women were specifically asked about symptoms associated with endometriosis such as dysmenorrhoea, chronic pelvic pain, dyspareunia, subfertility, dyschezia and cyclic rectal bleeding.

Transvaginal ultrasound examination was performed by two ultrasound operators who were both gynaecologists with a high level of expertise in gynaecological ultrasonography. The ultrasound operators were blinded to any previous surgical findings. All women were operated on by four different laparoscopic surgeons with a high level of expertise in laparoscopic surgery. The findings were recorded using the revised ASRM classification of the severity of endometriosis. When moderate, severe or deeply invasive disease (DIE) was present a complete surgical exploration of the pelvis was performed. This involved dissection of the pouch of Douglas when obliterated and resection of any DIE including the RVS, so as not to miss any disease. The operating surgeons were blinded to the detailed transvaginal ultrasound findings.

### Transvaginal ultrasound assessment of pelvic endometriosis

All women were examined in the dorsal lithotomy position using a high resolution transvaginal ultrasound probe. The examinations were performed in a standardised and systematic way. Firstly the uterus was assessed in the transverse and sagittal planes. Next the ovaries were found and their size was measured in three orthogonal planes.

Ovarian cysts were diagnosed as endometriomas when they appeared as well circumscribed thick walled cysts which contained homogenous low level internal echos (“ground glass”) [5]. Measurements were recorded from the inside of the cyst wall in three orthogonal planes. The average of the 3 diameters (D1 + D2 + D3)/3 was. The adnexa were also systematically examined for the presence of tubal dilatation.

Ovarian mobility was assessed by a combination of gentle pressure with the vaginal probe and abdominal pressure with the examiner’s free hand as in a bimanual examination. The ovary was deemed to be completely free when all of its borders could be seen sliding across the surrounding structures. Minimal adhesions were considered to be present when some of the surrounding structures could not be separated from the ovary with gentle pressure but the ovary could be mobilised from the majority (approximately >2/3) of the surrounding structures. Moderate adhesions were thought to be present when the ovarian mobility was reduced due to adhesions with the surrounding structures but the structures on 2/3-1/3 of the surface of the ovary were sliding across it on gentle pressure. Fixed ovaries could not be mobilised at all with gentle pressure nor separated from the surrounding structures. If the tubes were dilated, the mobility of the dilated tubes was documented in a similar fashion. Normal fallopian tubes are difficult to identify in the absence of background fluid in the pelvis and therefore it was not possible to score non dilated tubes for adhesions. It is difficult to see filmy adhesions on TVS unless there is fluid entrapped within the adhesions, giving rise to the “flapping sail sign” [6], or unless the mobility of the affected organs is reduced and therefore these features were not scored separately at TVS.

The presence of adhesions in the pouch of Douglas was assessed next. The uterus was gently mobilised by a combination of pressure on the cervix with the ultrasound probe alternating with pressure on the fundus from the examiners free hand on the abdominal wall. The aim was to watch the interface of the posterior uterine serosa and the bowel behind to ensure that the two structures were sliding easily across one another. If these two surfaces were completely free of one another this was assessed as no adhesions present. Complete obliteration was assessed as the absence of any sliding between the serosa on the posterior surface of the cervix or uterus and the bowel behind. Partial obliteration of the pouch of Douglas was present if there were some adhesions between the bowel and the uterus but some free sliding was seen. Partial obliteration was also present when adnexal structures were firmly adherent to the posterior aspect of the uterus but the bowel appeared to be free.

Endometriotic nodules or deeply invasive endometriosis (DIE) were typically visualised as stellate hypoechoic or isoechogenic solid masses with irregular outer margins [7,8], which were tender on palpation and fixed to the surrounding pelvic structures. They were usually located in the uterosacral ligaments, adnexa, rectovaginum, and urinary bladder. Endometriotic nodules located in the wall of the rectosigmoid colon tend to appear as hypoechoic thickenings of bowel muscularis propria, which sometimes protrude into the lumen of the bowel [9]. Rectovaginal endometriosis is defined as disease affecting the posterior pelvic compartment with evidence of endometriotic nodules which are located between the rectum and posterior fornix of the vagina and/or posterior aspect of the cervix. The presence and largest diameter of any deep lesions were documented.

All these findings were recorded on a database file using a Microsoft Excel for Windows spreadsheet to facilitate data entry and retrieval. The severity of endometriosis as assessed by TVS was compared with laparoscopic findings using the rASRM classification [10].

### Statistical analysis

All statistical analyses were carried out using Medcalc version 9.2.0.2 (Medcalc Software, Mariakerke, Belgium). The diagnostic accuracy of the tests was assessed using sensitivity, specificity, positive (PPV) and negative (NPV) predictive value, and positive (LR+) and negative (LR-) likelihood ratio measures. Overall levels of agreement for non binary data was calculated using Cohen’s quadratic weighted Kappa coefficient. Kappa values of 0.81-1.0 indicated very good agreement, Kappa values of 0.61-0.80 good agreement, Kappa values of 0.41-0.60 moderate agreement, Kappa values of 0.21-0.40 fair agreement and Kappa values <0.20 poor agreement [11,12]. The Kruskal-Wallis one-way analysis of variance was used to assess for statistical difference between rank sum of the groups as the data was not normally distributed.

## Results

From July 2006 to September 2009 we recruited 237 women into this study. 39 women were excluded from the final analysis: twenty nine because they were not assessed by one of the two designated ultrasound operators, five became pregnant whilst awaiting surgery, one cancelled her operation, one laparoscopy was unsuccessful and three women were lost to follow up.

198 women were included in the final analysis. The mean age was 35.0 (95% CI 33.98 – 35.97, SD 7.10) (range 19–50) years. The presenting symptoms were dysmenorrhoea for 143/198 (72.2%), chronic pelvic pain for 98/198 (49.5%), dyspareunia for 91/198 (45.9%), infertility for 42/198 (21.2%), dyschezia for 19/198 (9.6%) and cyclic rectal bleeding for 3/198 (1.5%) women. A single presenting symptom was present in 72/198 (36.4%) women, two presenting symptoms in 66/198 (33.3%), three presenting symptoms in 39/198 (19.7%), four or more symptoms in 19/198 (9.6%) women.

At laparoscopy 126/198 (63.6%) women had endometriosis. Of these women 30 /126 (23.8%) had stage 1 endometriosis by the rASRM classification, 24/126 (19.0%) had stage 2, 21/126 (16.7%) had stage 3 and 51/126 (40%) had stage 4 disease. Of the 104 women with focal lesions (excluding women with only diffuse superficial peritoneal disease) 28/104 (26.9%) women had endometriosis in a single location whilst the remaining 73.1% had endometriosis in two or more locations.

The ultrasound examinations were performed by two examiners: examiner A performed 104 (52.5%), examiner B 94 (47.5%). All women were operated on by one of four laparoscopic surgeons: surgeon A operated on 79 (39.9%), surgeon B on 54 (27.3%), surgeon C on 35 (17.7%) and surgeon D on 30 (15.2%) women. The mean interval between TVS and operation was 36.8 days (95% CI 33.4 – 41.1, SD 22.9) (range 0–87 days).

Table 1 shows the prevalence of the individual features of pelvic endometriosis at laparoscopy. Table 2 gives the details of the individual locations of endometriosis in relation to whether they were isolated lesions or multifocal lesions. Of the 104 women with focal lesions (excluding women with only diffuse superficial peritoneal disease) 28/104 (26.9%) of these women had endometriosis in a single location whilst the remaining 73.1% had endometriosis in two or more locations.

**Table 1:** The prevalence of endometriotic lesions at different anatomical locations at laparoscopy

**Site of disease**	**N (%)**
Endometrioma on either ovary	51/198 (25.7%)
Unilateral	27/198 (13.6%)
Bilateral	24/198 (12.1%)
Moderate/severe adhesions on either ovary	78/198 (39.4%)
Unilateral	30/198 (15.2%)
Bilateral	48/198 (24.2%)
DIE of USL unilateral	8/198 (4.0%)
DIE of USL bilateral	12/198 (6.1%)
Complete obilteration of POD	30/198 (15.2%)
Partial obilteration of POD	24/198 (12.1%)
DIE of Rectum/Sigmoid	11/198 (5.6%)
DIE of RVS	32/198 (16.2%)
DIE of bladder	5/198 (2.5%)
DIE of utero vesical fold (separate from bladder)	6/198 (3.0%)
DIE of PSW unilateral	7/198 (3.5%)
DIE of PSW bilateral	3/198 (1.5%)

DIE is deeply infiltrating endometriosis, USL is uterosacral ligaments, POD is pouch of Douglas, RVS is rectovaginal septum, PSW is pelvic side wall.

**Table 2:** Isolated and multiple endometriotic lesions in respect to their locations

**Site of disease**	**Endometriosis of a single location**	**Endometriosis multiple locations**
	**N (%)**	**N (%)**
Ovarian endometrioma n = 51	2/51 (3.9%)	49/51 (96.1%)
Ovarian adhesions n = 85	16/85 (18.8%)	69/85 (81.2%)
Adhesions in POD n = 54	1/54 (1.9%)	53/54 (98.1%)
USL DIE n = 23	5/23 (21.7%)	18/23 (88.3%)
RV or POD DIE n = 32	1/32 (3.1%)	31/32 (96.9%)
DIE of rectum or sigmoid n = 9	0/9 (0%)	9/9 (100%)
DIE of bladder n = 5	1/5 (20%)	4/5 (80%)
DIE of UVF n = 6	1/6 (16.7%)	5/6 (83.3%)
DIE of PSW n = 9	1/9 (11.1%)	8/9 (88.9%)
Total	28/104 (26.9%)	76/104 (73.1%)

DIE is deeply infiltrating endometriosis, USL is uterosacral ligaments, POD is pouch of Douglas, RVS is rectovaginal septum, UVF is utero vesical fold, PSW is pelvic side wall. All bilateral structures were considered as one location (ovaries, USL, PSW).

Ovarian endometriomas were rarely isolated lesions as ovarian adhesions were also present in 48/51 (94%) of cases. 27/51 (52.9%) women with endometriomas had unilateral and 24/51 (47.1%) had bilateral lesions. There was no significant difference in the frequency of endometriomas located in the right or left ovary (Chi-square =0.327 p = 0.51). Women with bilateral endometriomas were no more likely to have associated DIE 16/24 (66.6%) compared to women with unilateral endometriomas 14/27 (51.8%) (Chi-square =0.621 p = 0.431 stat).

Diagnostic accuracy of pre-operative TVS for each of the specific anatomical locations of endometriosis is shown in Table 3. There was a significant difference between the sensitivities for the different locations (Chi squared = 74.97, P < 0.0001) while the specificities were similar (p > 0.05). The positive likelihood ratio (LR+) was very useful (>10) for the TVS diagnosis of endometriosis of the following anatomical locations: ovarian endometriomas; moderate or severe ovarian adhesions; pouch of Douglas adhesions; and deeply infiltrating endometriosis (DIE) of the bladder; rectum or sigmoid; rectovaginum; uterovesical fold; and the uterosacral ligaments. Only for pelvic side wall DIE and mild ovarian adhesions was the LR + moderately useful (5–10). The negative likelihood ratio (LR-) was very useful (<0.1) for bladder DIE and moderately useful (0.1-0.2) for ovarian endometriomas, moderate or severe ovarian adhesions, and pouch of Douglas adhesions. The sensitivity was highest for bladder and ovarian endometriomas and lowest for DIE of the uterovasical fold, pelvic side wall and uterosacral ligaments.

**Table 3:** Accuracy of pre-operative ultrasound diagnosis of endometriotic lesions affecting different pelvic organs

**Site of disease**	**Sensitivity**	**Specificity**	**PPV**	**NPV**	**LR+**	**LR-**	**Area under ROC curve**
Ovarian endometrioma N = 75	84.0 (95% CI 73.7 – 91.4)	95.6(95% CI 92.8 – 97.6)	81.8 (95% CI 71.8 – 90.6)	96.2 (95% CI 93.5 – 97.8)	19.26 (95% CI 11.431 – 32.451)	0.167 (95% CI 0.10 – 0.281)	0.898 (95% CI 0.864 – 0.926)
							P = 0.0001
DIE of bladder N = 5	100 (95% CI 48.0 – 100)	100 (95% CI 98.1 – 100)	100 (95% CI 48.0 – 100)	100 (95% CI 98.1 – 100)	∞ (95% CI 0- ∞)	0.00 (95% CI 0- ∞)	1.00 (95% CI 0.981 – 1.00)
							P = 0.000
DIE Rectum/Sigmoid N = 9	33.3 (95% CI 12.1 – 64.6)	98.9 (95% CI 96.2 – 99.7)	60 (95% CI 23.1 – 88.2)	96.9 (95% CI 93.4 – 98.6)	31.5 (95% CI 5.992 – 165.6)	0.674 (95% CI 0.424 – 1.07)	0.661 (95% CI 0.591 – 0.727)
							P = 0.111
RV DIE N = 32	50.0 (95% CI 33.6 – 66.4)	100 (95% CI 97.7 – 100)	100 (95% CI 80.6 – 100)	96.9 (95% CI 93.4 – 98.6)	∞ (95% CI 0- ∞)	0.50 (95% CI 0.354 – 0.707)	0.758 (95% CI 0.692 – 0.816)
							P = 0.0001
DIE of UVF N = 6	16.7 (95% CI 2.8 – 63.9)	99.0 (95% CI 96.3 – 99.8)	33.3 (95% CI 6.1 – 79.2)	97.4 (95% CI 94.2 – 98.9)	16.0 (95% CI 1.68 – 153.94)	0.84 (95% CI 0.589 – 1.205)	0.578 (95% CI 0.506 – 0.648)
							P = 0.528
DIE of PSW N = 13	15.4 (95% CI 2.4 – 45.5)	98.17 (95% CI 96.3 – 99.3)	22.2 (95% CI 0.063 – 0.547)	97.2 (95% CI 95.0 – 98.4)	8.421 (95% CI 1.933 – 36.65)	0.862 (95% CI 0.683 – 1.087)	0.568 (95% CI 0.517 – 0.617)
							P = 0.419
DIE of USL N = 40	10.0 (95% CI 2.9 – 23.7)	99.16(95% CI 97.6 – 99.8)	57.1 (95% CI 25.0 – 84.2)	90.7 (95% CI 87.5 – 93.2)	11.867 (95% CI 2.754 – 51.14)	0.908 (95% CI 0.818 – 1.007)	0.546 (95% CI 0.495 – 0.596)
							P = 0.351

PPV is positive predictive value, NPV is negative predictive value, +ve LH is positive likelihood ratio, -ve LH is negative likelihood ratio, ROC is receiver operating characteristics, DIE is deeply infiltrating endometriosis, RV is rectovaginal, UVF is utero vesical fold (separate from bladder), PSW is pelvic side wall, USL is uterosacral ligaments. (All bilateral anatomical locations were treated separately).

**Figure 1:**
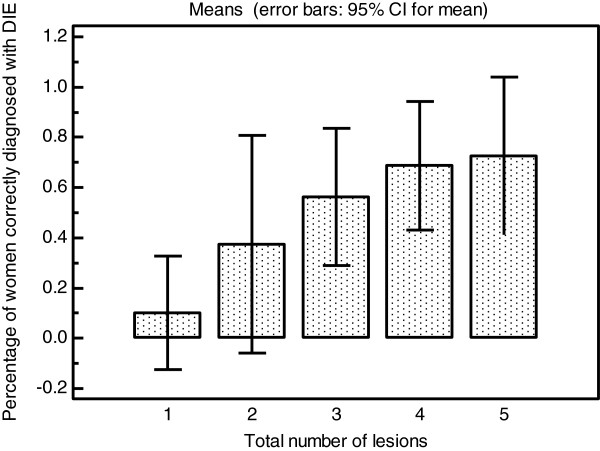
Bar chart of total number of endometriotic lesions at laparoscopy against percentage of women correctly diagnosed with DIE in each group.

**Figure 2:**
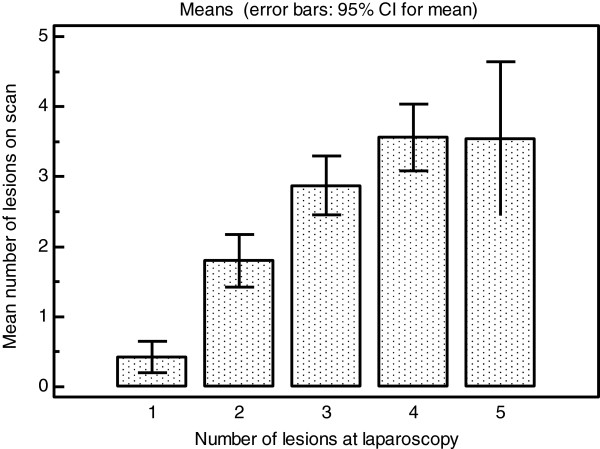
Bar chart of total number of endometriotic lesions at laparoscopy against the mean number of lesions seen on scan in each group.

**Figure 3:**
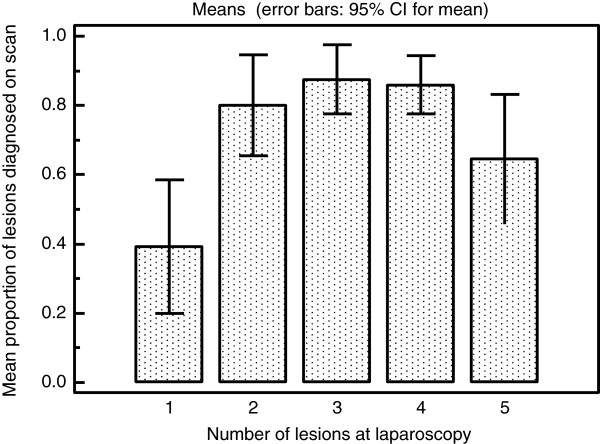
Bar chart of total number of endometriotic lesions seen at laparoscopy against mean proportion of lesions diagnosed on scan in each group.

The LR + and –LR for all adhesions on the ovaries were moderately and somewhat useful respectively. However for the assessment of moderate or severe adhesions on the ovary the LR + and –LR was very and moderately useful respectively as detailed in Table 4. When the diagnosis of ovarian adhesions was stratified according to the ASRM classification into mild, moderate and severe the overall level of agreement between scan and laparoscopy was very good (Table 5). The LR + and –LR for adhesions in the pouch of Douglas were very and moderately useful respectively as detailed in Table 4. When pouch of Douglas obliteration was assessed according to the ASRM classification into partial and complete obliteration the overall level of agreement between scan and laparoscopy was very good (Table 6). Table 7 shows that the accuracy of the diagnosis of DIE increases significantly with the total number of endometriotic lesions present. This data is represented graphically in Figure 1. Table 8 shows that although the number of endometriotic lesions seen on scan significantly increases with the number of lesions present (Figure 2) the proportion of the total lesions correctly diagnosed increases to a maximum at three lesions present at laparoscopy then declines (Figure 3).

**Table 4:** Accuracy of pre-operative ultrasound diagnosis of pelvic adhesions in women with suspected endometriosis

**Site of disease**	**Sensitivity**	**Specificity**	**PPV**	**NPV**	**LR+**	**LR-**	**Area under ROC curve**
Any adhesions on ovary N = 130	79.6 (95% CI 72.0 – 85.5)	91.9 (95% CI 87.9 – 94.6)	83.8 (95% CI 76.6 – 89.2)	89.5 (95% CI 85.2 – 92.6)	9.81 (95% CI 6.456 – 14.92)	0.222 (95% CI 0.160 – 0.310)	0.865 (95% CI 0.827 – 0.897)
							P = 0.0001
Mod/Severe adhesions on ovary N = 123	83.7 (95% CI 76.2 – 89.2)	94.1 (95% CI 90.7 – 96.4)	86.6 (95% CI 79.3 – 91.6)	92.8 (95% CI 89.1 – 95.3)	14.288 (95% CI 8.826 – 23.131)	0.173 (95% CI 0.116 – 0.258)	0.889 (95% CI 0.854 – 0.919)
							P = 0.0001
Severe adhesions on ovary N = 103	83.5 (95% CI 75.1 – 89.4)	93.5 (95% CI 90.1 – 95.8)	81.9 (95% CI 73.5)	94.2 (95% CI 90.8 – 96.3)	12.876 (95% CI 8.266 – 20.057)	0.176 (95% CI 0.114 – 0.273)	0.867 (95% CI 0.830 – 0.899)
							P = 0.0001
Any adhesions in POD N = 54	83.3 (95% CI 71.3 – 91.0 )	95.1 (95% CI 90.3 – 97.6)	86.5 (95% CI 74.7 – 93.3)	93.8 (95% CI 88.7 – 96.7)	17.143 (95% CI 8.242 – 35.656)	0.175 (95% CI 0.096 – 0.318)	0.892 (95% CI 0.841 – 0.932)
							P = 0.0001
Complete obliteration of POD N = 30	83.3 (95% CI 66.4 – 0.927)	97.0 (95% CI 93.2 – 98.7)	83.3 (95% CI 66.4 – 92.7)	97.0 (95% CI 93.2 – 98.7)	28.0 (95% CI 11.636 – 67.376)	0.172 (95% CI 0.077 – 0.383)	0.902 (95% CI 0.852 – 0.939)
							P = 0.0001

PPV is positive predictive value, NPV is negative predictive value, +ve LH is positive likelihood ratio, -ve LH is negative likelihood ratio, ROC is receiver operating characteristics, POD is pouch of Douglas.

**Table 5:** Comparison of ultrasound and laparoscopy for the assessment of severity of ovarian adhesions

	**TVS assessment of ovarian adhesions**				
**Laparoscopic assessment of ovarian adhesions**	**Absent**	**Minimal**	**Moderate**	**Severe**	**Total**
Absent	238	6	5	10	259 (65.4%)
Minimal	10	3	0	1	14 (3.5%)
Moderate	7	1	4	8	20 (5.1%)
Severe	11	1	5	86	103 (26.0%)
Total	266 (67.2%)	11 (2.8%)	14 (3.5%)	105 (26.5%)	396

Weighted Kappa = 0.801 (standard error (Kw’ = 0) 0.050 and (Kw’#0) 0.031).

**Table 6:** Comparison of ultrasound and laparoscopy for the assessment of severity of adhesions in the pouch of Douglas

	**Pouch of Douglas obliteration at TVS**			
**Pouch of Douglas obliteration at laparoscopy**	**No adhesions**	**Partial obliteration**	**Complete obliteration**	**Total**
No adhesions	137	4	3	144 (72.7%)
Partial obliteration	9	13	2	24 (12.1%)
Complete obliteration	0	5	25	30 (15.2%)
Total	146 (73.7%)	22 (11.1%)	30 (15.2%)	198

Weighted Kappa kappa = 0.852 (standard error (Kw’ = 0) 0.071 and (Kw'#0) 0.038).

**Table 7:** Women with DIE separated into groups by total number of endometriotic lesions compared with the accuracy of diagnosis of DIE in each group

**Total number of endometriotic lesions**	**Number of women (n = 61)**	**Number correctly diagnosed with DIE (n,%)**
Single lesions	10	1 (10.0%)
2 lesions	8	3 (37.5%)
3 lesions	16	9 (56.3%)
4 lesions	16	11 (68.8%)
5 lesions or more	11	8 (72.7%)

Kruskal-Wallis test of correlation between total number of lesions and% of women correctly identified with DIE (P = 0.0228).

**Table 8:** Shows the mean number and mean proportion of lesions diagnosed on scan for all women with endometriotic lesions grouped by total number of lesions

**Total number of lesions**	**Number of women N = 104**	**Mean number of lesions diagnosed on scan**	**Mean proportion of total lesions diagnosed on scan**
Single lesions	28	0.429 (95% CI 0.207 to 0.651)	0.3929 (95% CI 0.2000 to 0.5857)
2 lesions	25	1.800 (95% CI 1.4232 to 2.1768)	0.8000 (95% CI 0.6541 to 0.9459)
3 lesions	24	2.8750 (95% CI 2.4562 to 3.2938)	0.8750 (95% CI 0.7749 to 0.9751)
4 lesions	16	3.5625 (95% CI 3.0871 to 4.0379)	0.8594 (95% CI 0.7756 to 0.9432)
5 lesions or more	11	3.5455 (95% CI 2.4471 to 4.6438)	0.6450 (95% CI 0.4584 to 0.8316)
		P < 0.0001*	P = 0.0008*

*Kruskal-Wallis test of correlation between total number of lesions at laparoscopy and mean number of lesions diagnosed on scan and mean proportion of total lesions diagnosed on scan respectively.

## Discussion

Our study has shown that pre-operative transvaginal ultrasound examination can be used to diagnose pelvic endometriosis and to assess its severity. The total number of endometriotic lesions found at laparoscopy has statistically significant positive effect on the accuracy of ultrasound diagnosis of deeply infiltrating lesions. The sensitivity of the ultrasound diagnosis was significantly affected by the location of the endometriotic lesions but the specificity remained high throughout. We have shown for the first time that ultrasound enables detection and assessment of severity of adhesions affecting the ovaries and pouch of Douglas.

The accuracy of TVS was highest in the diagnosis of ovarian adhesions, pouch of Douglas obliteration and bladder lesions. The accuracy for these features was similar to the accuracy for ovarian endometriomas which were previously thought of as the only feature of pelvic endometriosis which it is possible to diagnose on ultrasound [1]. Previous studies have stated that left sided endometriomas are more common than right [13] but there was no statistically significant difference in our data set. Our study has also shown that only 26.9% of women with focal endometriosis will have disease in only one location and therefore in all cases the examiner should perform a detailed search for lesions in other typical locations.

There are few studies on the accuracy of TVS for the diagnosis of ovarian adhesions. Our study has shown a high level of accuracy for this diagnosis with a kappa value of 0.801. No study has previously assessed severity of ovarian adhesions classified as either minimal, moderate or severe in accordance with the rASRM classification [14]. Guerriero et al., [15] used the combination of three features as suggestive of ovarian adhesions: blurring of the ovarian margin, the inability to mobilise the ovary on palpation (fixation) and an increased distance from the probe. They found that these tests either combined or individually gave a kappa value of between 0.25 and 0.51. Okaro et al., [2] examined women with chronic pelvic pain prior to laparoscopy for the presence of ovarian adhesions and classified them as either mobile or fixed. They found a high degree of agreement between TVS and laparoscopy at identifying ovarian adhesions (0.81 kappa). This compares with the results of our study of an area under the ROC of 0.889 for the presence of either moderate or severe adhesions and a kappa of 0.801 for the three stages of severity. Yazbek et al., [16] examined the role of ultrasound for the preoperative assessment of adnexal masses. They found a sensitivity of 44% and a specificity of 98% in the diagnosis of severe pelvic adhesions. The technique for examination of adhesions was similar to that used in this paper but they do not state ovarian adhesions separately. Guerriero et al., [3] used a technique of applying pressure between the uterus and ovary. If they remained linked then this was suggestive of adhesions. This gave a sensitivity and specificity of 89% and 90% respectively for fixation of the ovaries to the uterus.

The preoperative diagnosis of partial or complete obliteration of the pouch of Douglas has not been reported on directly before. Our study shows a high accuracy of this diagnosis. Hudelist [17] gave a high accuracy for the diagnosis of pouch of Douglas endometriosis but did not report obliteration separately. Yazbek [16] described the technique for diagnosing POD obliteration but did not report this finding separately from severe pelvic adhesions.

The high level of accuracy for the diagnosis of bladder endometriosis is concordant with previous studies, which showed a high level of accuracy in the TVS diagnosis of bladder endometriosis [7,8].

There were poor levels of sensitivity for the diagnosis of endometriosis affecting the uterosacral ligaments and pelvic side walls. The low accuracy of TVS for diagnosing endometriosis of the uterosacral ligaments and pelvic side walls has also been previously reported [18,19]. Hudelist et al., [20] report higher levels of sensitivity for the diagnosis of uterosacral disease however these levels were lower than for almost all of the other locations of DIE. The preoperative diagnosis of endometriosis in these locations is not critical for the management as these are rarely missed at laparoscopy and surgical excision can usually be achieved without involvement of other surgical specialists.

Our study showed a high specificity of the diagnosis of rectovaginal disease and a lower sensitivity. This agrees with the results of a recent review by Hudelist [21] encompassing 10 studies on the diagnostic accuracy of TVS for intestinal endometriosis. He found sensitivities ranging from 67- 98% and specificites of 92-100%.

The effect of the number of lesions on the sensitivity of ultrasound diagnosis of specific endometriotic lesions in different locations has not been assessed before. Our data shows that the accuracy of the diagnosis of individual specific lesions increases with their absolute number up to a maximum of three lesions. With increasing number of lesions above that level the sensitivity declines. A possible reason for this could be that in more severe disease the adhesions tend to obscure other small lesions further away from the ultrasound probe. There is also a possibility of operator bias as in women with evidence of severe disease documentation of the presence of small lesions such as those located at utero-sacral ligaments becomes less clinically relevant.

Our study could be criticised for not more accurately differentiating between DIE of the rectum and sigmoid or between rectovaginal and vaginal disease. We could also be criticised for including subjective assessments such as ovarian and pouch of Douglas mobility which cannot be recorded with ease. However we diagnosed ovarian and pouch of Douglas disease with greater accuracy than other features of endometriosis which indicates that subjective assessment is accurate enough to be used in routine practice. Reproducibility of these findings however needs to be externally validated before we can reach a consensus about the value of subjective assessment for the diagnosis of ovarian and pouch of Douglas adhesions. Scanning for endometriosis is difficult and we believe that the use of palpation is of critical importance to achieve good diagnostic accuracy. Gynaecologists use palpation routinely as part of pelvic examination and they can incorporate it more easily into ultrasound examination than sonographers or radiologists. For this reason it remains to be seen whether these results can be extrapolated to units with different levels of experience and expertise.

The benefit of an accurate diagnosis of individual features of endometriosis is that it provides a better overall assessment of the severity of the disease and aids in counselling and planning of treatment. If surgery is required, then women with severe disease may be referred to a tertiary centre with expertise in treating bladder and bowel disease. Prior knowledge of the extent of the disease facilitates comparisons of clinical symptoms with anatomical locations of endometriotic lesions. This improves pre-operative counselling of women and helps to tailor treatment in a way which will ensure excision of symptomatic lesions and avoid complex procedures to remove asymptomatic lesions from difficult anatomical locations. It also aids the surgeon in planning the operation and ensuring that the necessary staff are available, such as colorectal surgeons, when treatment of the disease involving bowel is required. Preoperative underestimation of the severity of DIE lesions increases the risk of incomplete surgical excision, further progression of the residual disease and the need for multiple surgical procedures [22,23].

## Conclusions

Our study has shown that the specificity of the ultrasound diagnosis of pelvic endometriotic lesions is high with low false positive rates. The negative diagnostic rate was less high especially in the diagnosis of bowel, rectovaginal, uterosacral ligament, pelvic side wall and uterosacral ligament lesions. Therefore women with significant symptoms and a negative diagnosis still require further investigation. The accuracy of ultrasound diagnosis is significantly affected by the location and number of endometriotic lesions.

### Details of ethics approval

Kings College Hospital, London, Research Ethics Committee reference number 06/Q0703/119. Full title of study. The accuracy of gynaecological ultrasound examination for the diagnosis of severe pelvic endometriosis.

## Abbreviations

ASRM: American society of reproductive medicine; CI: Confidence interval; DIE: Deeply infiltrating endometriosis; PPV: Positive predictive value; NPV: Negative predictive value; LR+: Positive likelihood ratio; LR-: Negative likelihood ratio; SD: Standard deviation.

## Competing interests

ES received honoraria from Ethicon for provision of training to healthcare professionals and consultancy fees from Bayer. AC is on the advisory board for surgical innovations for which he receives an annual honorarium. AC also received support for courses and education from Storz and Johnson and Johnson and support for clinical nursing from Covidien and Lotus. The other authors declared no competing interests.

## Authors’ contributions

TH designed the study protocol, wrote the ethics committee application, recruited and scanned approximately half the patients, collected data, analysed the data and drafted the manuscript. AC and ES operated on many patients and collected data. DM and KP collected data. DJ conceived of the study, and participated in its design and coordination, recruited and scanned approximately half the patients and helped with data analysis. All authors revised the manuscript and read and approved the final manuscript.

## Pre-publication history

The pre-publication history for this paper can be accessed here:

http://www.biomedcentral.com/1472-6874/13/43/prepub
